# 5-Phenyl-1,3,4-oxadiazol-2-amine

**DOI:** 10.1107/S1600536812040640

**Published:** 2012-09-29

**Authors:** Man-Man Song, Kong-Li Wu, Lin Zhu, Juan Zheng, Yan Xu

**Affiliations:** a Department of Chemistry, Zhengzhou University, Zhengzhou, 450052, People’s Republic of China

## Abstract

In the title complex, C_8_H_7_N_3_O, the C—O [1.369 (2) and 1.364 (3) Å] and C=N [1.285 (3) and 1.289 (3) Å] bond lengths in the oxadiazole ring are each almost identical within systematic errors, although different substituents are attached to the ring. The phenyl ring is inclined to the planar oxadiazole ring [r.m.s. deviation 0.002 Å] by 13.42 (18)°. In the crystal, molecules are linked *via* N—H⋯N hydrogen bonds, forming double-stranded chains propagating along [010].

## Related literature

For background to 5-phenyl-1,3,4-oxadiazol-2-amines and the synthesis of the title compound, see: Bachwani *et al.* (2011 ▶); Lv *et al.* (2010 ▶); Tang *et al.* (2007 ▶).
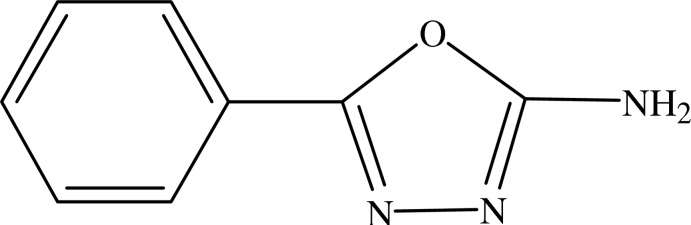


## Experimental

### 

#### Crystal data

C_8_H_7_N_3_O
*M* *_r_* = 161.17
Monoclinic, 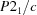

*a* = 11.194 (3) Å
*b* = 5.8990 (5) Å
*c* = 15.034 (5) Å
β = 130.193 (18)°
*V* = 758.3 (3) Å^3^
*Z* = 4
Mo *K*α radiation
μ = 0.10 mm^−1^
*T* = 291 K
0.26 × 0.24 × 0.22 mm

#### Data collection

Agilent Xcalibur Eos Gemini diffractometer
Absorption correction: multi-scan (*CrysAlis PRO*; Agilent, 2011 ▶) *T*_min_ = 0.851, *T*_max_ = 1.000
2912 measured reflections
1551 independent reflections
877 reflections with *I* > 2σ(*I*)
*R*_int_ = 0.040

#### Refinement

*R*[*F* ^2^ > 2σ(*F* ^2^)] = 0.056
*wR*(*F* ^2^) = 0.137
*S* = 1.02
1551 reflections
109 parameters
H-atom parameters constrained
Δρ_max_ = 0.17 e Å^−3^
Δρ_min_ = −0.15 e Å^−3^

### 

Data collection: *CrysAlis PRO* (Agilent, 2011 ▶); cell refinement: *CrysAlis PRO*; data reduction: *CrysAlis PRO*; program(s) used to solve structure: *SHELXS97* (Sheldrick, 2008 ▶); program(s) used to refine structure: *SHELXL97* (Sheldrick, 2008 ▶); molecular graphics: *OLEX2* (Dolomanov *et al.*, 2009 ▶); software used to prepare material for publication: *OLEX2*.

## Supplementary Material

Crystal structure: contains datablock(s) global, I. DOI: 10.1107/S1600536812040640/im2386sup1.cif

Structure factors: contains datablock(s) I. DOI: 10.1107/S1600536812040640/im2386Isup2.hkl

Additional supplementary materials:  crystallographic information; 3D view; checkCIF report

## Figures and Tables

**Table 1:** Hydrogen-bond geometry (Å, °)

*D*—H⋯*A*	*D*—H	H⋯*A*	*D*⋯*A*	*D*—H⋯*A*
N3—H3*A*⋯N2^i^	0.89	2.12	2.997 (3)	169
N3—H3*B*⋯N1^ii^	0.95	2.12	3.054 (3)	168

Symmetry codes: (i) 
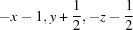
; (ii) 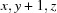.

**Fig. 1.**
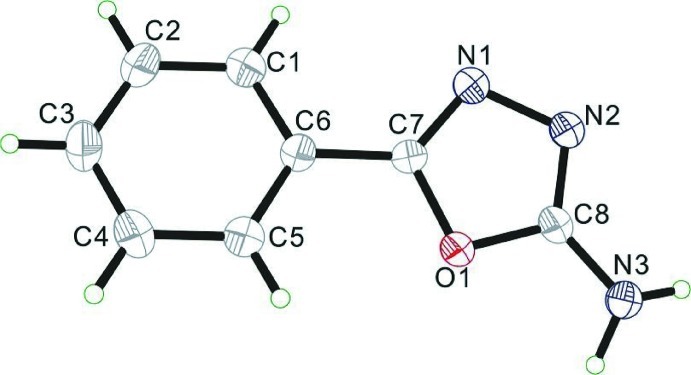
View of the title compound showing thermal ellipsoids at the 30% probability level. H atoms are omitted for clarity.

**Fig. 2.**
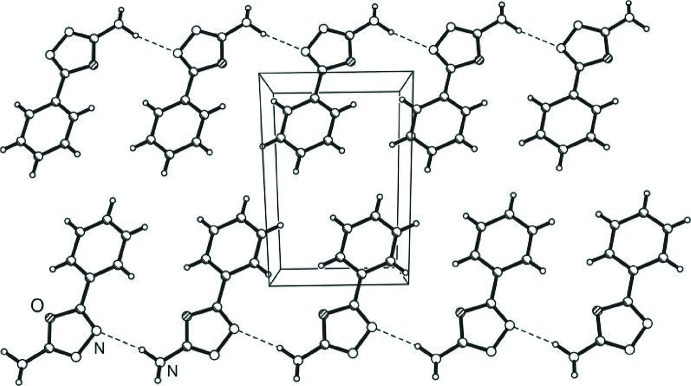
View of the title complex, showing the hydrogen bonding in the crystal structure.

## supplementary crystallographic information

### Comment

Oxadiazole is the parent compound for a vast class of heterocyclic compounds. Oxadiazole derivatives have attracted considerable attention (Bachwani *et al.*, 2011). Although 1,3,4-oxadiazole exhibit various N and O atoms that should allow to form single crystals due to their ability to act as hydrogen bond acceptor sites, there have been limited studies concerning their crystal properties. To further explore their crystal properties, in this communication, we report the crystal structure of the title compound. The molecular structure of the title compound is shown in Fig. 1. As shown in figure 1, the bond length between the O1 with C7 is nearly to the bond length between the O1 with C8, they are 1.369 (2) Å and 1.364 (3) Å. Similarly, the distance of the bond between the C7 and N1 is 1.285 (3) Å and the distance of the bond between the C8 and N2 is 1.289 (3) Å. The bond length between N1 with N2 is 1.413 (3) Å and the bond length between N3 with C8 is 1.328 (3) Å. In the crystal structure, the C7—N1—N2 and C8—N2—N1 angles are 106.97 (18) ° and 105.75 (19) °. The torsion angle between C(7)—N(1)—N(2)—C(8) is 0.2 (3) ° demonstrating the planarity of the heterocyclic moiety. Classical intermolecular N(1)—H···N(3) (3.054 Å) and N(2)—H···N(3) (2.997 Å) hydrogen bonds link the adjacent molecules into a two-dimensional structure.

### Experimental

Benzaldehyde (0.01 mol) and ethanol (20 ml) were added to semicarbazide hydrochloride (0.011 mol) and the reaction mixture was refluxed for 2 h. Afterwards the obtained semicarbazone (0.01 mol) was dissolved in acetic acid together with bromine (0.65 ml) and the solution was stirred for 30 minutes. The resulting precipitate (0.02 mmol) was dissolved in a ethanol (3 ml) water (3 ml) mixture. The resulting solution was allowed to stand at room temperature for about two weeks days. Colourless crystals were obtained in a yield of 43%.

### Refinement

All H atoms are positioned geometrically with C—H = 0.93 Å and N—H = 0.95 Å and refined as riding atoms with *U*_iso_(H) = 1.2*U*_eq_(C,N).

### Crystal data

**Table:**

C_8_H_7_N_3_O	*F*(000) = 336
*M**_r_* = 161.17	*D*_x_ = 1.412 Mg m^−^^3^
Monoclinic, *P*2_1_/*c*	Mo *K*α radiation, λ = 0.7107 Å
*a* = 11.194 (3) Å	Cell parameters from 656 reflections
*b* = 5.8990 (5) Å	θ = 3.5–26.3°
*c* = 15.034 (5) Å	µ = 0.10 mm^−^^1^
β = 130.193 (18)°	*T* = 291 K
*V* = 758.3 (3) Å^3^	Prism, colourless
*Z* = 4	0.26 × 0.24 × 0.22 mm

### Data collection

**Table:**

Agilent Xcalibur Eos Gemini diffractometer	1551 independent reflections
Radiation source: Enhance (Mo) X-ray Source	877 reflections with *I* > 2σ(*I*)
Graphite monochromator	*R*_int_ = 0.040
Detector resolution: 16.2312 pixels mm^-1^	θ_max_ = 26.4°, θ_min_ = 3.6°
ω scans	*h* = −13→12
Absorption correction: multi-scan (*CrysAlis PRO*; Agilent, 2011)	*k* = −4→7
*T*_min_ = 0.851, *T*_max_ = 1.000	*l* = −18→17
2912 measured reflections	

### Refinement

**Table:**

Refinement on *F*^2^	Primary atom site location: structure-invariant direct methods
Least-squares matrix: full	Secondary atom site location: difference Fourier map
*R*[*F*^2^ > 2σ(*F*^2^)] = 0.056	Hydrogen site location: inferred from neighbouring sites
*wR*(*F*^2^) = 0.137	H-atom parameters constrained
*S* = 1.02	*w* = 1/[σ^2^(*F*_o_^2^) + (0.054*P*)^2^] where *P* = (*F*_o_^2^ + 2*F*_c_^2^)/3
1551 reflections	(Δ/σ)_max_ < 0.001
109 parameters	Δρ_max_ = 0.17 e Å^−^^3^
0 restraints	Δρ_min_ = −0.15 e Å^−^^3^

### Special details

**Table:**

Experimental. CrysAlisPro, Agilent Technologies, Version 1.171.35.19 (release 27-10-2011 CrysAlis171 .NET) (compiled Oct 27 2011,15:02:11) Empirical absorption correction using spherical harmonics, implemented in SCALE3 ABSPACK scaling algorithm.
Geometry. All e.s.d.'s (except the e.s.d. in the dihedral angle between two l.s. planes) are estimated using the full covariance matrix. The cell e.s.d.'s are taken into account individually in the estimation of e.s.d.'s in distances, angles and torsion angles; correlations between e.s.d.'s in cell parameters are only used when they are defined by crystal symmetry. An approximate (isotropic) treatment of cell e.s.d.'s is used for estimating e.s.d.'s involving l.s. planes.
Refinement. Refinement of *F*^2^ against ALL reflections. The weighted *R*-factor *wR* and goodness of fit *S* are based on *F*^2^, conventional *R*-factors *R* are based on *F*, with *F* set to zero for negative *F*^2^. The threshold expression of *F*^2^ > σ(*F*^2^) is used only for calculating *R*-factors(gt) *etc*. and is not relevant to the choice of reflections for refinement. *R*-factors based on *F*^2^ are statistically about twice as large as those based on *F*, and *R*- factors based on ALL data will be even larger.

### Fractional atomic coordinates and isotropic or equivalent isotropic displacement parameters (Å^2^)

**Table:**

	*x*	*y*	*z*	*U*_iso_*/*U*_eq_	
O1	−0.15541 (17)	0.6145 (2)	0.02276 (13)	0.0495 (5)	
N1	−0.2177 (2)	0.2637 (3)	−0.04164 (18)	0.0617 (7)	
N2	−0.3435 (2)	0.4062 (3)	−0.12624 (17)	0.0594 (7)	
N3	−0.3795 (2)	0.8025 (3)	−0.12550 (17)	0.0622 (7)	
H3A	−0.4628	0.8128	−0.2012	0.075*	
H3B	−0.3161	0.9341	−0.0922	0.075*	
C1	0.1056 (3)	0.1191 (4)	0.1680 (2)	0.0624 (8)	
H1	0.0506	0.0125	0.1084	0.075*	
C2	0.2512 (3)	0.0674 (4)	0.2723 (3)	0.0683 (9)	
H2	0.2942	−0.0749	0.2825	0.082*	
C3	0.3329 (3)	0.2221 (5)	0.3606 (2)	0.0714 (9)	
H3	0.4308	0.1850	0.4303	0.086*	
C4	0.2704 (3)	0.4311 (5)	0.3463 (2)	0.0720 (9)	
H4	0.3259	0.5365	0.4065	0.086*	
C5	0.1250 (3)	0.4866 (4)	0.2428 (2)	0.0578 (8)	
H5	0.0828	0.6292	0.2336	0.069*	
C6	0.0423 (3)	0.3320 (4)	0.1532 (2)	0.0457 (6)	
C7	−0.1115 (3)	0.3912 (4)	0.0434 (2)	0.0450 (6)	
C8	−0.3014 (3)	0.6083 (4)	−0.0839 (2)	0.0477 (7)	

### Atomic displacement parameters (Å^2^)

**Table:**

	*U*^11^	*U*^22^	*U*^33^	*U*^12^	*U*^13^	*U*^23^
O1	0.0394 (10)	0.0366 (9)	0.0426 (9)	−0.0023 (7)	0.0129 (8)	−0.0024 (7)
N1	0.0457 (13)	0.0409 (11)	0.0551 (13)	−0.0007 (10)	0.0129 (11)	−0.0039 (10)
N2	0.0413 (12)	0.0417 (12)	0.0529 (13)	−0.0017 (10)	0.0112 (11)	−0.0038 (10)
N3	0.0462 (13)	0.0409 (12)	0.0514 (13)	0.0025 (10)	0.0097 (11)	0.0036 (10)
C1	0.0511 (17)	0.0476 (15)	0.0623 (17)	−0.0008 (13)	0.0247 (15)	−0.0049 (13)
C2	0.0508 (17)	0.0531 (16)	0.0730 (19)	0.0128 (14)	0.0273 (16)	0.0128 (14)
C3	0.0463 (16)	0.075 (2)	0.0537 (18)	0.0022 (15)	0.0143 (14)	0.0111 (15)
C4	0.0611 (19)	0.067 (2)	0.0468 (16)	−0.0069 (16)	0.0164 (15)	−0.0053 (14)
C5	0.0522 (17)	0.0457 (14)	0.0529 (16)	0.0005 (12)	0.0236 (15)	−0.0019 (12)
C6	0.0391 (14)	0.0415 (13)	0.0467 (14)	0.0012 (11)	0.0232 (12)	0.0037 (11)
C7	0.0404 (14)	0.0330 (12)	0.0487 (14)	−0.0002 (11)	0.0229 (12)	0.0003 (11)
C8	0.0365 (14)	0.0447 (14)	0.0422 (13)	−0.0044 (12)	0.0164 (12)	−0.0025 (12)

### Geometric parameters (Å, º)

**Table:**

O1—C7	1.369 (2)	C1—C6	1.387 (3)
O1—C8	1.364 (3)	C2—H2	0.9300
N1—N2	1.413 (3)	C2—C3	1.366 (4)
N1—C7	1.285 (3)	C3—H3	0.9300
N2—C8	1.289 (3)	C3—C4	1.364 (4)
N3—H3A	0.8936	C4—H4	0.9300
N3—H3B	0.9476	C4—C5	1.382 (3)
N3—C8	1.328 (3)	C5—H5	0.9300
C1—H1	0.9300	C5—C6	1.376 (3)
C1—C2	1.382 (3)	C6—C7	1.464 (3)
			
C8—O1—C7	102.89 (16)	C3—C4—H4	119.9
C7—N1—N2	106.97 (18)	C3—C4—C5	120.2 (2)
C8—N2—N1	105.75 (18)	C5—C4—H4	119.9
H3A—N3—H3B	115.3	C4—C5—H5	119.8
C8—N3—H3A	119.2	C6—C5—C4	120.3 (2)
C8—N3—H3B	114.6	C6—C5—H5	119.8
C2—C1—H1	120.4	C1—C6—C7	120.3 (2)
C2—C1—C6	119.2 (2)	C5—C6—C1	119.5 (2)
C6—C1—H1	120.4	C5—C6—C7	120.3 (2)
C1—C2—H2	119.5	O1—C7—C6	118.30 (19)
C3—C2—C1	121.0 (2)	N1—C7—O1	111.77 (19)
C3—C2—H2	119.5	N1—C7—C6	129.9 (2)
C2—C3—H3	120.1	N2—C8—O1	112.6 (2)
C4—C3—C2	119.8 (3)	N2—C8—N3	130.2 (2)
C4—C3—H3	120.1	N3—C8—O1	117.12 (19)
			
N1—N2—C8—O1	0.7 (3)	C4—C5—C6—C1	0.5 (4)
N1—N2—C8—N3	−176.1 (3)	C4—C5—C6—C7	−179.8 (2)
N2—N1—C7—O1	−0.4 (3)	C5—C6—C7—O1	13.9 (3)
N2—N1—C7—C6	−180.0 (2)	C5—C6—C7—N1	−166.5 (3)
C1—C2—C3—C4	0.1 (5)	C6—C1—C2—C3	0.3 (4)
C1—C6—C7—O1	−166.5 (2)	C7—O1—C8—N2	−0.9 (3)
C1—C6—C7—N1	13.1 (4)	C7—O1—C8—N3	176.4 (2)
C2—C1—C6—C5	−0.5 (4)	C7—N1—N2—C8	−0.2 (3)
C2—C1—C6—C7	179.8 (3)	C8—O1—C7—N1	0.8 (3)
C2—C3—C4—C5	−0.1 (5)	C8—O1—C7—C6	−179.6 (2)
C3—C4—C5—C6	−0.2 (4)		

### Hydrogen-bond geometry (Å, º)

**Table:**

*D*—H···*A*	*D*—H	H···*A*	*D*···*A*	*D*—H···*A*
N3—H3*A*···N2^i^	0.89	2.12	2.997 (3)	169
N3—H3*B*···N1^ii^	0.95	2.12	3.054 (3)	168

Symmetry codes: (i) −*x*−1, *y*+1/2, −*z*−1/2; (ii) *x*, *y*+1, *z*.
